# Patient-specific placental vessel segmentation with limited data

**DOI:** 10.1007/s11701-024-01981-z

**Published:** 2024-06-04

**Authors:** Gary Sarwin, Jonas Lussi, Simone Gervasoni, Ueli Moehrlen, Nicole Ochsenbein, Bradley J. Nelson

**Affiliations:** 1 Computer Vision Lab, ETH Zurich, 8092 Zurich, Switzerland; 2 Multi-Scale Robotics Lab, ETH Zurich, 8092 Zurich, Switzerland; 3 Department of Obstetrics, University Hospital of Zurich, 8091 Zurich, Switzerland; 4 Department of Pediatric Surgery, University Children’s Hospital Zurich, 8032 Zurich, Switzerland; 5 Zurich Center for Fetal Diagnosis and Therapy, University of Zurich, 8006 Zurich, Switzerland

**Keywords:** Generative adversarial networks, Medical image generation, Segmentation, Twin-to-twin transfusion syndrome

## Abstract

A major obstacle in applying machine learning for medical fields is the disparity between the data distribution of the training images and the data encountered in clinics. This phenomenon can be explained by inconsistent acquisition techniques and large variations across the patient spectrum. The result is poor translation of the trained models to the clinic, which limits their implementation in medical practice. Patient-specific trained networks could provide a potential solution. Although patient-specific approaches are usually infeasible because of the expenses associated with on-the-fly labeling, the use of generative adversarial networks enables this approach. This study proposes a patient-specific approach based on generative adversarial networks. In the presented training pipeline, the user trains a patient-specific segmentation network with extremely limited data which is supplemented with artificial samples generated by generative adversarial models. This approach is demonstrated in endoscopic video data captured during fetoscopic laser coagulation, a procedure used for treating twin-to-twin transfusion syndrome by ablating the placental blood vessels. Compared to a standard deep learning segmentation approach, the pipeline was able to achieve an intersection over union score of 0.60 using only 20 annotated images compared to 100 images using a standard approach. Furthermore, training with 20 annotated images without the use of the pipeline achieves an intersection over union score of 0.30, which, therefore, corresponds to a 100% increase in performance when incorporating the pipeline. A pipeline using GANs was used to generate artificial data which supplements the real data, this allows patient-specific training of a segmentation network. We show that artificial images generated using GANs significantly improve performance in vessel segmentation and that training patient-specific models can be a viable solution to bring automated vessel segmentation to the clinic.

## Introduction

Deep learning applications in the medical field have experienced considerable success in both research and practice in recent years [28]. However, translating these techniques to clinics faces challenges due to the scarcity of high-quality datasets, resulting from the cost and time involved in their creation. Data distribution disparities between training data and clinical practice commonly occur due to diverse acquisition techniques and patient variances. Additionally, limited dataset sizes, often from a single facility within a specific timeframe, may introduce bias, hindering model generalization to other facilities and impeding implementation in medical practice [30].

To tackle these challenges, we propose a patient-specific approach that trains a neural network in-situ using data from a single patient. Unlike standard training pipelines requiring numerous samples, we utilize generative adversarial networks (GANs) in a customized data processing pipeline.

We present a segmentation network trained on a limited dataset with the aid of data generated by GANs. Leveraging GANs enables us to create artificial data that augments more comprehensively than traditional methods like flipping and rotating. Our pipeline employs multiple GANs to automatically produce additional labeled training data, enriching the segmentation network’s understanding of variability within the original dataset. We use a progressively growing GAN (PGGAN) to generate masks, which are then fed into a conditional GAN (cGAN) to create artificial images. These synthetic images are added to the real dataset to train the segmentation network. We demonstrate this pipeline for patient-specific placental vessel segmentation, achieving promising results with as few as 20 images collected and labeled on-site by an expert.

**Fig. 1:**
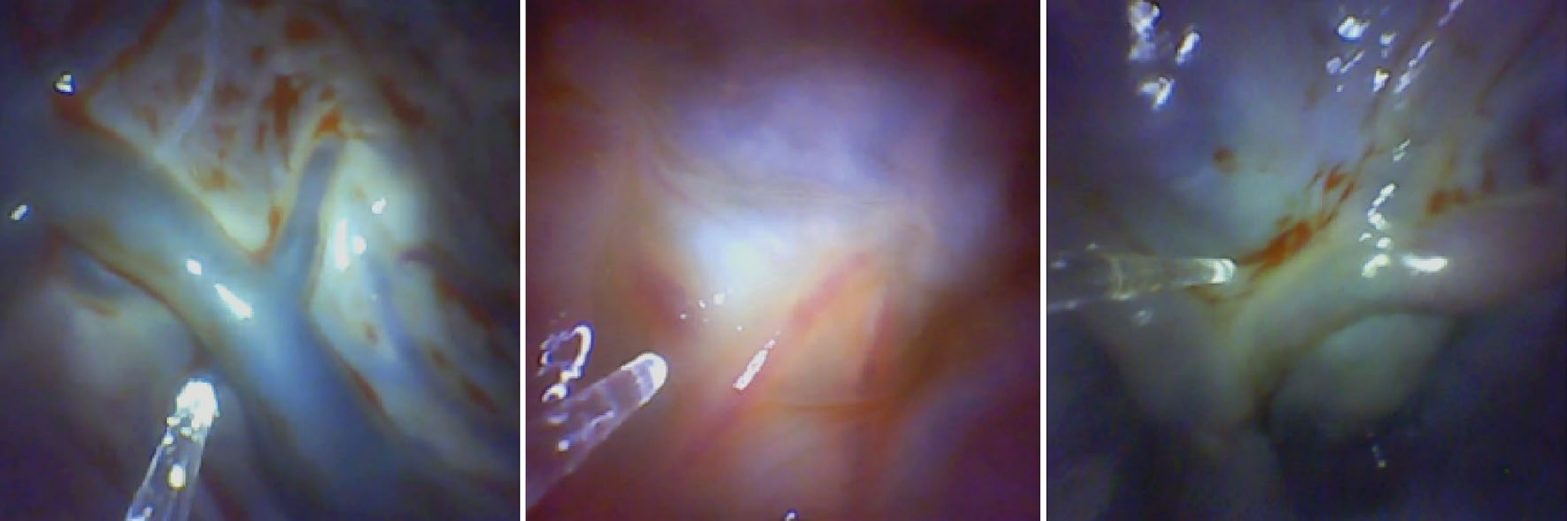
This figure illustrates substantial variations in placental vessels among patients, posing challenges when deploying a model and encountering significant differences between training and inference data

### Placental Vessel Segmentation

The challenge of variation across patients arises in placental imaging with a specialized endoscope (Fig. 1). Unlike a one-size-fits-all model, a patient-specific network can address this distribution shift. Previously deemed infeasible due to on-site data labeling requirements, recent advances in learning-based methods, particularly GANs, offer a solution, paving the way for new applications. For instance, patient-specific vessel segmentation could aid clinicians during fetoscopic laser coagulation, a procedure for treating twin-to-twin transfusion syndrome (TTTS) [3].

TTTS, a rare complication of monochorionic twin pregnancies, affects twins sharing a single placenta. It results from abnormal vascular connections that disproportionately transfer blood from one twin (the donor) to the other (the recipient). If untreated, TTTS can lead to severe complications, including cardiac issues, long-term neurodevelopmental impairment, and even death for one or both twins [3, 32]. Various treatment options exist, with fetoscopic laser coagulation proving superior in most cases [1]. This minimally invasive procedure employs a fetoscope with a laser tool to coagulate abnormal vascular connections on the placental chorionic plate [3].

### Motivation

Fetoscopic laser coagulation has reduced complications, but challenges persist due to the lack of effective pre-surgery imaging of abnormal vasculature. Imaging during the procedure is hindered by amniotic fluid opacity, limited fetoscope view, occlusions, and poor illumination. These obstacles can impede blood vessel identification, leading to incomplete ablation and persistent Twin-to-twin transfusion syndrome (TTTS). Complete ablation is crucial for successful treatment. Segmentation of the vasculature is a vital step toward providing surgeons with a comprehensive view to identify all problematic blood vessels and ensure successful TTTS treatment [3, 27].

## Related work

### Vessel segmentation

Automatic detection of vascular structures facilitates the process of determining which vessels are to be ablated by the surgeon. Blood vessel analysis is important for diagnosis and treatment in many medical fields, such as ophthalmology [5], oncology, and neurosurgery [23].

Moccia et al. [23] reviewed various state-of-the-art approaches for automatic vessel segmentation, and methods based on deep learning are now favored due to advances in the field. Although artificial intelligence in the medical field is gaining more traction, a significant limitation is the lack of high-quality datasets. One of the more explored areas is the field of ophthalmology because of the availability of the STARE [15] and DRIVE [29] datasets, which have resulted in a wide range of methods to tackle the problem of retinal blood vessel segmentation.

For cases with limited data, compelling approaches have been proposed such as creating synthetic data for vessel segmentation using only a single annotated image [26], which greatly reduces labeling efforts, but requires long training times. Additionally, style-transfer has been proposed as well using going from X-ray angiograms to vessel segmentation without the need for any labeled angiograms [24]. Finally, methods have been proposed using automatically generated labels from multimodal data or using weak supervision with pseudo-labels [14, 33, 34]. These methods have shown promising results but rely on either multiple modalities or clear distinguishable edges which works well in modalities such as X-ray images or retinography. However, this could become problematic in endoscopic procedures where lighting conditions and the surroundings are challenging.

Scientific output regarding placental vessel segmentation during TTTS treatment is scarce. Gaisser et al. [10] suggested the use of deep convolutional neural networks (CNN) for panorama reconstruction, which yielded superior results compared to feature extraction methods in an in vivo setting. However, this method, which is based on a single-shot multibox detector, did not perform well under the varying lighting conditions during TTTS treatment. Sadda et al. [27] devised a U-Net [25] architecture on 345 frames labeled by a clinician. The trained U-Net performed better than other filtering methods, even outperforming a novice labeler. In 2020, Bano et al. [2] released the first publicly available labeled placenta dataset that contained 483 images and segmentation masks, with various U-Net architectures on the dataset to compare the results. All tested architectures achieved better results than the network used by Sadda et al. The best-performing model was the U-Net with a ResNet101 [17] backbone.

### GANs

Generative adversarial networks were first introduced in 2014 by Goodfellow et al. [12], who proposed a new framework for estimating generative models via an adversarial process. Two networks are trained in this process; a generator G, which captures the data distribution, and a discriminator D, which estimates with probability whether a sample comes from the training data or is produced by the generator G. The alternating consecutive training of both networks allows the GAN to address complicated generative problems. Since their introduction, GANs have been widely used in research, and an incredible amount of variation in their architecture has been proposed. In the medical field, one application frequently explored is domain adaptation that allows switching from one image modality to another, for example from magnetic resonance (MR) to computed tomography (CT) images [20], or when labels of only one domain are available and sought after in another, as shown in [6].

**Fig. 2:**
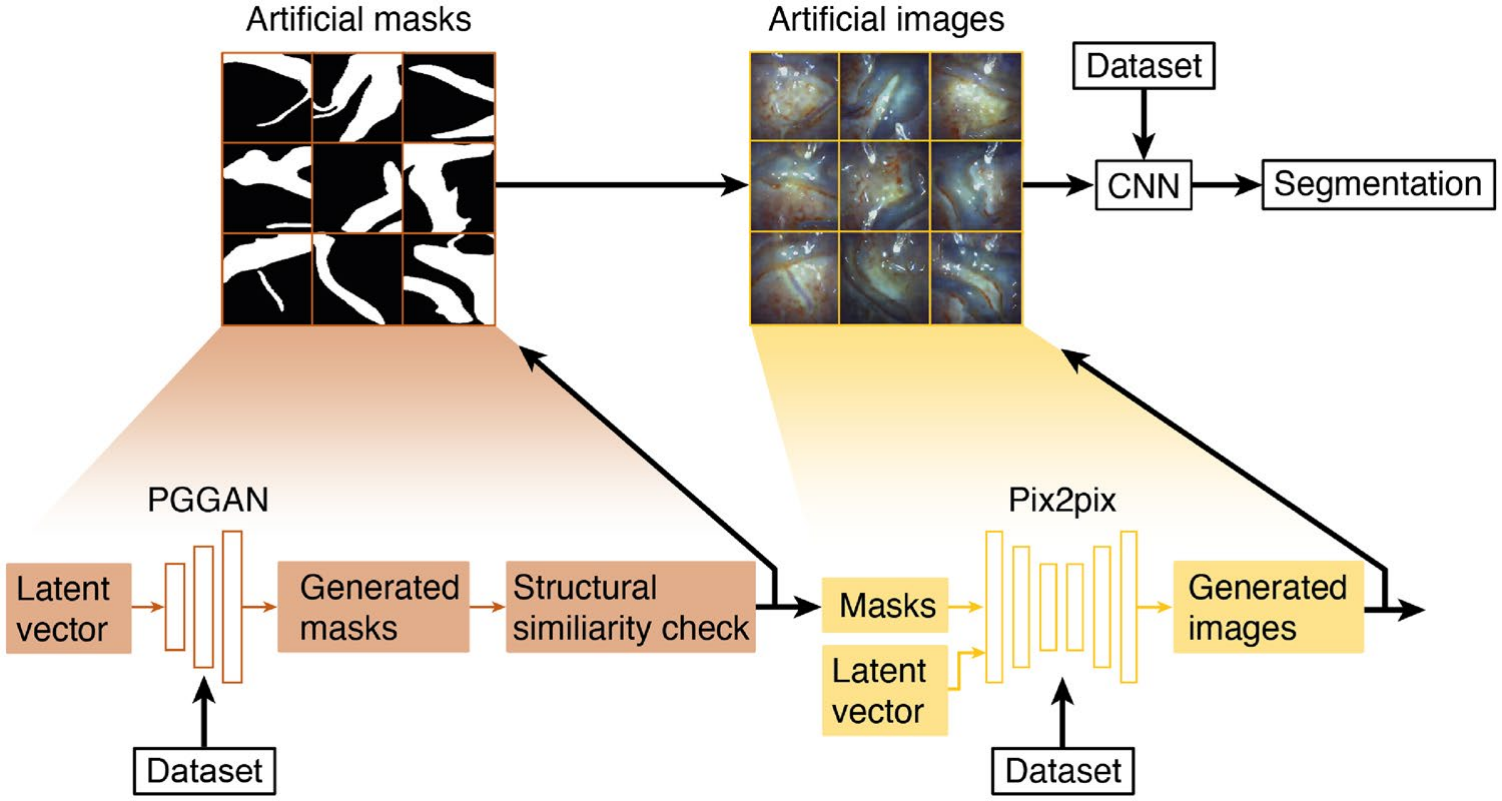
A PGGAN takes a sampled latent variable as input and produces masks. After a structural similarity check, the masks are inputted into the pix2pix network to generate placental images. These image-mask pairs, along with the original dataset used to train the PGGAN and pix2pix networks, were then utilized to train a segmentation network

### Generation of synthetic images

Another application of GANs is generating synthetic images or data for training subsequent networks, as demonstrated in this study. This concept has been explored previously, such as for retinal images [8] and liver lesion classification by generating artificial lesions [9].

Unlike processing MRI and CT images, where appearance remains relatively consistent across patients, fetoscopic images exhibit significant variation among patients. Consequently, we adapted the GAN augmentation pipeline and training using generated images for a patient-specific approach.

## Methods

This study focuses on creating realistic fetoscopic placental images through image-to-image translation using data from a single patient to train a segmentation network. The proposed pipeline involves multiple components, detailed in Fig. 2. Initially, a PGGAN generates binary masks, which are then translated into placental images. These generated image-mask pairs are utilized to train the segmentation network. The subsequent section elaborates on the generation of masks and corresponding placental images, along with their utilization in training the network for vessel segmentation.

### Mask generation

For TTTS applications, a stable network that can generate realistic masks with only a few training samples is required. Therefore, a progressively growing GAN was used to generate masks. Progressive growth enables stable learning from coarse to fine details, such as the branching of placental vessels. During this training method, both the generator and the discriminator grow progressively. Specifically, both start with low resolution, thereby capturing coarse structures. As training progresses, additional layers are added to the networks to model increasingly fine details over the course of the training to increase speed and training stability. The two GANs have similar but mirrored structures while maintaining all trainable layers during training. [18]

The implementation and training scheme presented by [7] were used. This implementation was selected to make the presented pipeline readily available for implementation by third adopters without any significant modifications. Standard image augmentation, such as flipping and rotation, was also applied to the training images to help avoid mode collapse. The structural similarity between the generated masks was computed to filter out images that were very similar to one another. All masks were compared and if a structural similarity above 90% was calculated, the mask was discarded. The combination of augmentation and the structural similarity check yielded sufficiently varying and realistic masks that were useful for further processing using as little as 20 training images.

The training was performed in 6 stages, doubling the initial 4 $\times$ 4 resolution in every stage to finally reach an output of 256 $\times$ 256 pixels, using Wasserstein loss and gradient norm as loss (WGAN-GP) [13]. The use of this loss function changes the discriminator into a critic while allowing for more stable training, often eliminating the need for hyperparameter tuning. The last sigmoid layer of the discriminator is removed and thereby changes it from a probability to a value function *f* and introduces a gradient norm penalty, producing the following objective

$$\begin{aligned} \mathcal {L}=\underbrace{\underset{\tilde{\varvec{x}} \sim \mathbb {P}_g}{\mathbb {E}}\left[ D(\tilde{\varvec{x}})\right] -\underset{\varvec{x} \sim \mathbb {P}_r}{\mathbb {E}}\left[ D(\varvec{x})\right] }_{\text {Critic loss }}\\ +\underbrace{\lambda \underset{\hat{\varvec{x}} \sim \mathbb {P}_{\hat{\varvec{x}}}}{\mathbb {E}}\left[ \left( \left\| \nabla _{\hat{\varvec{x}}} D(\hat{\varvec{x}})\right\| _{2}-1\right) ^2\right] }_{\text {Gradient penalty}}, \end{aligned}$$

which consists of two terms, the critic loss, and the gradient penalty. In the first term $\varvec{x}$ denotes a real image, $\tilde{\varvec{x}}$ denotes a generated image from a sampled latent variable, $\lambda$ denotes the gradient penalty coefficient, and $\mathbb {P}_r$ and $\mathbb {P}_g$ denote the real data distribution and the generated distribution, respectively. In the second term, $\hat{\varvec{x}}$ denotes a random sample from $\mathbb {P}_{\hat{\varvec{x}}}$, which is implicitly defined sampling uniformly along straight lines between pairs of points sampled from the data distribution $\mathbb {P}_r$ and the generator distribution $\mathbb {P}_g$.

### Image generation

The established pix2pix network [16] was employed for image generation, learning the mapping from a binary vessel mask to the desired endoscopic placental image. Specifically, pix2pix is a conditional GAN (cGAN) learning the mapping from an observed image *x* and a random noise vector *z* to *y*, the output image, denoted as $G: \{x, z\} \rightarrow y$ [16]. Paired images of masks and placental images were necessary to train the segmentation network; thus, a cGAN was used to generate placental images. Consequently, the GAN is conditioned by the segmentation mask from the PGGAN, resulting in a matching-generated image-and-mask pair. The objective of a conditional GAN can be expressed as

$$\begin{aligned} \mathcal {L}(G, D) = \mathbb {E}_{x, y}{\left[ \textrm{log}\,D(x,y)\right] } + \mathbb {E}_{x, z}{\left[ \textrm{log}(1-D(x, G(x,z)))\right] }. \end{aligned}$$

The generator *G* tries to minimize this objective against an adversarial discriminator D that tries to maximize it. In combination with an L1 distance

$$\begin{aligned} \mathcal {L}_{L1}(G) = \mathbb {E}_{x, y, z}{\left[ ||y-G(x, z)||_1\right] }, \end{aligned}$$

which encourages the generator’s output to be close to the target image instead of only trying to trick the discriminator, the final objective of the pix2pix network is

$$\begin{aligned} G^* = \arg \underset{G}{\min }\,\underset{D}{\max }\,\mathcal {L}_{cGAN}(G, D) + \lambda \mathcal {L}_{L1}(G). \end{aligned}$$

This network offers the advantage of learning both the mapping and the loss function required for training, providing a versatile solution applicable to diverse problems. This eliminates the necessity for parameter adjustments and manual design of the loss function. The pix2pix implementation utilized adheres to the standard PyTorch implementation provided by [16], employing the default training scheme.

### Segmentation

A feature pyramid network [21] architecture with an EffiecientNetB5 [31] backbone was selected for the segmentation task. To conduct the training, both the generated images and real images were used. Different numbers of generated images with respect to the real images were used for the experiments, as explained in Sect. 4. Standard image augmentation techniques were applied, such as random flipping, rotating, and zooming, irrespective of the nature of the data: real or generated. The network was trained using the Adam [19] optimizer, Jaccard loss, and the weights that performed best on the validation set with respect to the intersection over union (IoU) were used. An initial learning rate of $3 \times 10^{-4}$ was used and reduced by a factor of 10 when the validation IoU metric did not increase over the previous 5 epochs.

## Experiments & results & discussion

Initially, an experiment investigated the potential performance enhancement of the segmentation network using the generated data for training. This experiment considered two parameters: the initial dataset size of the real data used for training both the GANs and the segmentation network, and the ratio of generated data to real data for training the segmentation network.

Subsequently, another experiment aimed to determine the superiority of the patient-specific approach compared to a network trained on a larger dataset but not on the specific patient. All experiments were conducted using a TITAN Xp GPU (Nvidia, USA).

### Dataset

#### MSRL dataset

The dataset used, was recorded using a fetoscope developed by the Multi-Scale Robotics Lab at ETH [11, 22]. The dataset was created in an ex vivo setting, where a human placenta was placed in an opaque container to simulate the dark environment of the human body encountered during surgery. The tip of the laser ablation tool was always visible in the images. The human placentas were acquired with written consent from patients and with approval from the Ethical Committee of the District of Zurich (study Stv22/2006). The dataset consists of images from three different placentas with a total of 513 annotated images coming from a single video per placenta. In the first experiment, the “Ratio Experiment,^1^” the images of the first placenta, the “training placenta”, were used, which amounted to 269 images in total. These images of the training placenta were randomly divided into sets of 163, 53, and 53 images, which represent the training, validation and test set, respectively. In the second experiment, the "Pre-training Experiment^2^", the vessel segmentation network was trained on the images of the second and third placentas, the "pre-training placentas", consisting of 244 images. These placentas are placentas from different patients than the training placenta to simulate an environment where a model has been trained on various patients and is deployed on unseen data from a different patient. The network was tested on the test set of the training placenta. The images were recorded with a 400 $\times$ 380 pixels resolution and the frames were cropped to 256 $\times$ 256 pixels for further processing.

#### UCL dataset

To validate the results achieved with the MSRL dataset, experiments were also performed on the dataset released by [2], the UCL dataset. This dataset consists of 483 annotated images from six different in vivo surgeries.

### Synthetic image generation

**Fig. 3:**
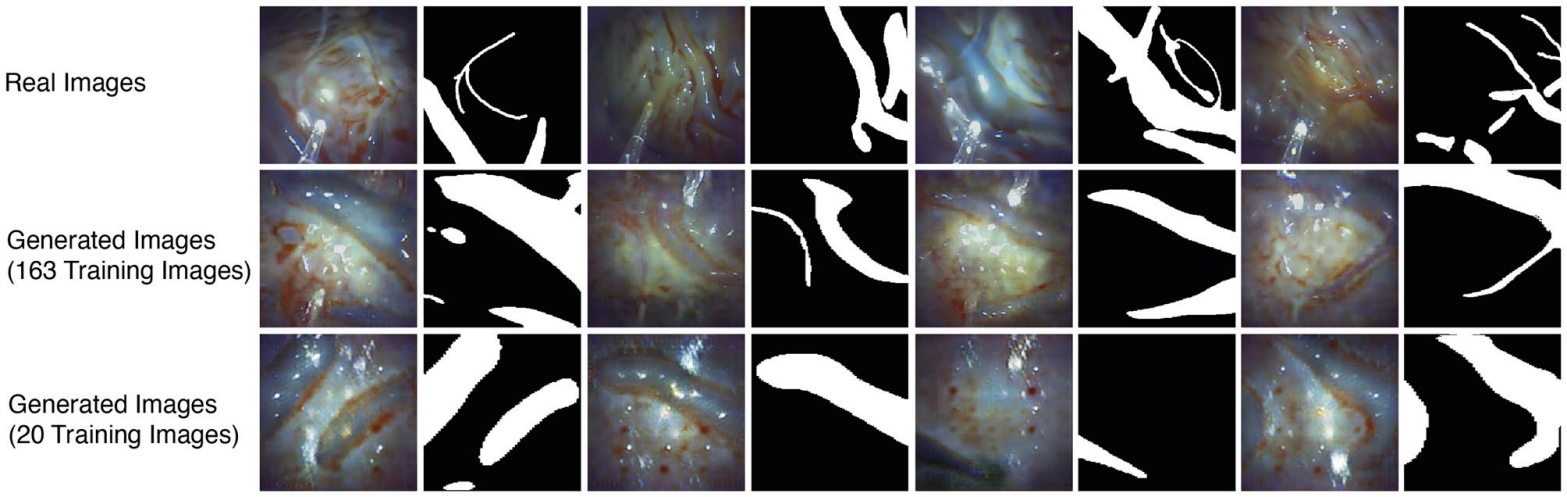
Examples of generated image pairs. Comparison between the real image and mask pairs and generated image pairs from the GANs, with varying real training dataset sizes

Real images with corresponding vessel masks are depicted in Fig. 3, alongside images and masks generated by the pipeline trained on 163 and 20 images, respectively. Additional generated image-and-mask pairs are available in Appendix A. Upon inspection, it is evident that the PGGAN can generate vessels of varying diameters. The pipeline trained on 163 images exhibits a wider range in vessel diameters overall and within a single mask, compared to the pipeline trained on 20 images, where vessels resemble blobs in multiple examples.

Analyzing the generated images from the pix2pix network reveals a close resemblance to real images in terms of overall appearance, color, and light reflection. A comparison between networks trained on 163 and 20 images demonstrates that the former better fills in placenta areas devoid of vessels, while the latter produces repetitive patches in these intervessel regions.

The evaluation of the quality of generated images is a well-known problem [4]. Assessing the usefulness of the generated images for a segmentation network and the captured images of the training data is challenging. Although the generated images may not show perfect images of a placenta, they do show vessels under different and challenging conditions, which could be sufficient to achieve a performance increase in segmentation.

### Placental vessel segmentation

#### Ratio experiment

**Fig. 4:**
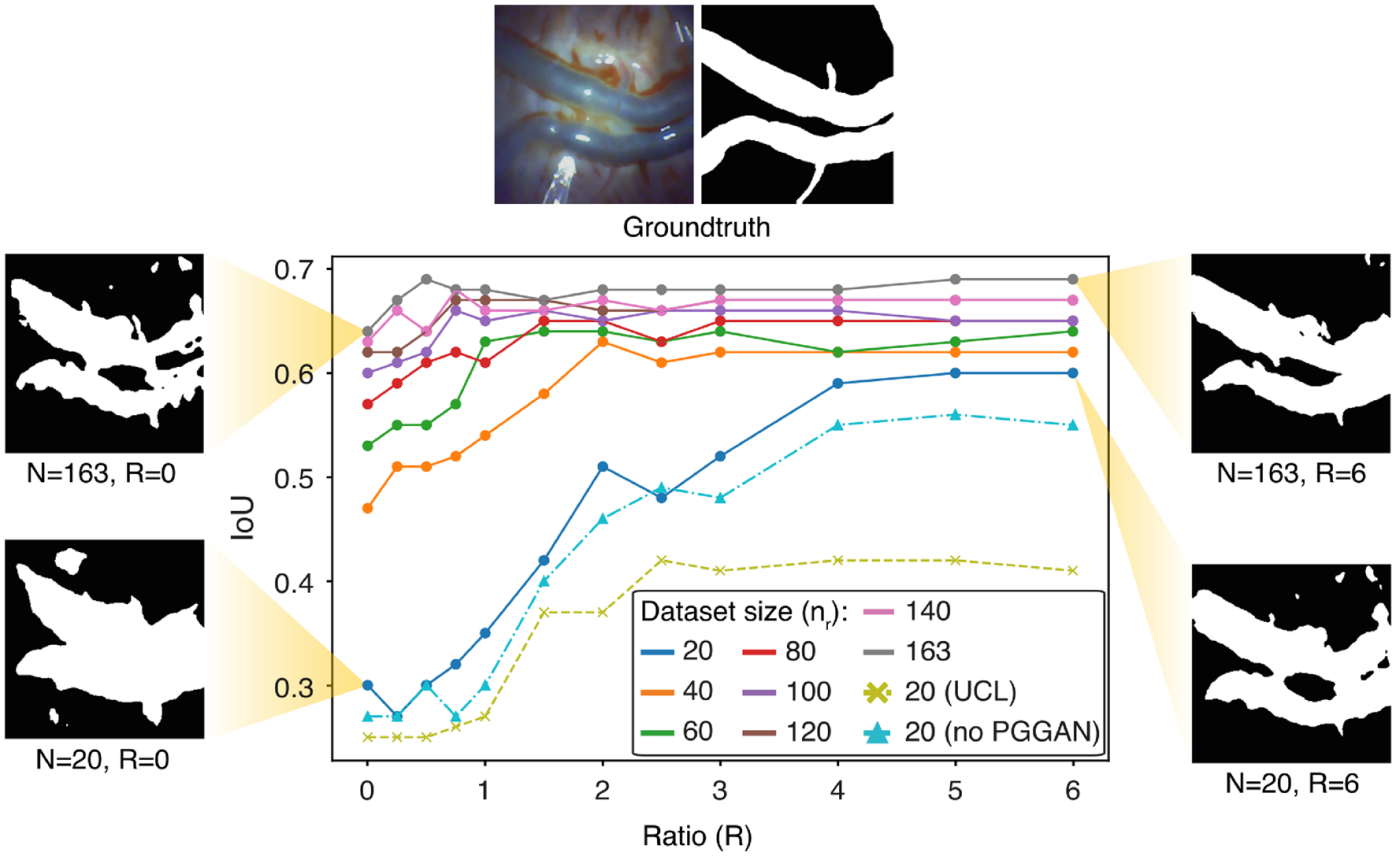
Experimental results. The segmentation performance of the ratio experiment is shown here for different initial dataset sizes as well as for different ratios of artificial and real data. A significant performance improvement was achieved using the generated data, especially for the initial dataset size of 20 images. The improvements range from 8 to 100%. Additionally, the results are shown for the pipeline applied to another dataset (UCL), as well as the results discussed in Sect. 4.4 for inference, without a patient-specific trained PGGAN (no PGGAN)

An insightful parameter to consider for the following experiment is the ratio *R*:

1 $$\begin{aligned} R = \frac{n_\textrm{artificial}}{n_\textrm{real}}, \end{aligned}$$

which is the amount of artificial data divided by the amount of real data, as well as the available dataset $n_\textrm{real}$ used for training. This experiment aims to explore the effects of various R and $n_\textrm{real}$ on the segmentation performance. Multiple experiments were conducted to validate the performance of the pipeline with various combinations of $n_\textrm{artificial}$ and $n_\textrm{real}$. Specifically, the training placenta from the MSRL dataset was split into training, validation, and testing splits of sizes 163, 53, and 53, respectively. The training images were randomly subsampled five times for every $n_\textrm{real}\in (20,40,60,80,100,120,140)$, and once for $n_\textrm{real}=163$ as this is the total size of the training dataset. This resulted in 35+1 subsets that were individually used to train the PGGAN and the pix2pix networks. Subsequently, the artificial images and labels were merged with the respective subsets to train the segmentation network.

Different numbers of artificial images were added to the respective subset to obtain various *R*. For every $R\in ~(0,0.25,0.5,0.75,1,1.5,2,2.5,3,4,5,6)$, three independent trials were performed in which the generated data combined with the real data was varied for each trial.

To evaluate the performance on the test set, the best-performing model of each subset across three different trials for every combination with *R* was selected. This resulted in five CNNs for every combination of $n_\textrm{real}$ and *R*. These models were then used to predict the test set. The results are presented in Fig. 4. Each data point consists of an average of five trained networks. The only exception is for $n_\textrm{real} = 163$, we did not subsample here as it would have resulted in the same subset five times. Therefore, the data points for that specific $n_\textrm{real}$ consist of only one trained network.

As can be seen in Fig. 4 and Table 1, significant performance increases were gained using artificial data, especially for the subsets of size 20. The mean increase in performance was 100% for $n_\textrm{real}=20$, going from an IoU score of 0.30 at $R=0$ to 0.60 at $R=6$. The achievable increase in performance decreased with an increasing dataset size. Furthermore, the ratio at which performance increase stagnates also achieved lower values with increasing dataset sizes. Notably, the segmentation of the networks at $n_\textrm{real}=20$ and $R=6$ achieved a similar IoU score to the segmentation of the networks at $n_\textrm{real}=163$ and $R=0$.

The results of the subset size of 20 images are particularly interesting because they require the least amount of labeling and achieve the greatest performance increase. Therefore, to validate the results, the same method was applied to the UCL dataset [2], where we performed five trials using 20 images that were randomly selected and trained the entire pipeline with it. The average IoU score increased from 0.25 to a maximum 0.42 at $R=2.5$, which corresponds to a performance increase of 68%, as shown in Fig. 4 (UCL). Although the increase is less than that achieved on the MSRL dataset, it is still very significant and shows that the pipeline fares well even for footage created from different instruments and institutions.

**Table 1:** Results. The results of ratio experiment are shown here together with the IoU for $R=0$ and the highest IoU achieved for each subset using additional synthetic data for training

Dataset Size	IoU at $R=0$	Std Augment	GAN Max IoU	Increase
20	0.30	0.30	0.60 ($R_\textrm{min}=5$)	100%
40	0.47	0.49	0.63 ($R_\textrm{min}=2$)	34%
60	0.53	0.53	0.64 ($R_\textrm{min}=1.5$)	21%
80	0.57	0.56	0.65 ($R_\textrm{min}=1.5$)	14%
100	0.60	0.58	0.66 ($R_\textrm{min}=0.75$)	10%
120	0.62	0.60	0.67 ($R_\textrm{min}=0.75$)	8%
140	0.63	0.61	0.68 ($R_\textrm{min}=0.75$)	8%
163	0.64	0.61	0.69 ($R_\textrm{min}=0.5$)	8%

Additionally, for the highest IoU, the smallest R is indicated that reached a certain IoU. Furthermore, the results for standard augmentation techniques are shown

#### Pre-training experiment

To assess the superiority of the patient-specific approach over a network previously trained on other patients, a segmentation network was trained on pre-training placentas. The pre-training dataset comprised 244 images, with 207 used for training and 37 for validation. This network achieved an IoU of 0.44 on the test set from the training placenta, inferior to any results achieved by the presented GANs-based pipeline for any initial dataset size. This supports the viability of the patient-specific approach.

#### Comparison to standard augmentation

To compare with standard augmentation techniques, an additional experiment was conducted across various dataset sizes. Following the same protocol as the ratio experiment but for $R=0$, random augmentations of brightness, contrast, saturation, and hue were applied alongside standard augmentations. Table 1 presents the average results on the test set. While random augmentations often improve performance, the intricate nature of placental images may limit such gains. In many cases, we observed no improvement or even a decrease in performance. This could be attributed to the diverse colors, textures, etc., seen across patients, where each combination is highly patient-specific.

### Inference

Some adjustments are necessary to implement the proposed pipeline in a surgical setting. While the pix2pix and segmentation networks converge within minutes, the PGGAN requires several hours. However, comparable results can be achieved using a PGGAN trained on a different placenta. To validate this, the ratio experiment was repeated with a dataset of 20 images. The PGGAN was trained using 20 randomly selected masks from pre-training placentas, while the pix2pix and segmentation networks were trained using corresponding images and masks from the training placenta. This process was repeated five times as in the "Ratio Experiment", resulting in an IoU increase from 0.27 to 0.56 at $R=5$, a 107% increase (Fig. 4, no PGGAN). This experiment demonstrates that the PGGAN does not need to be retrained for each patient, significantly reducing the pipeline’s implementation time for specific patients, rendering it suitable for surgical procedures. The total training time for the pix2pix network over 200 epochs is around 130 s, and the segmentation network converges after 15 epochs, taking approximately 70 s on an A100 GPU (Nvidia, USA). Thus, the total training time is under 3.5 min, allowing for on-site training within the 10-min exploration phase at the beginning of the procedure to assess fetal alignment and placental vessel structure.

## Conclusion

In this study, we introduced an intraoperative approach using limited patient-specific data to train a segmentation network for identifying placental blood vessels, addressing the distribution shift problem. GANs were utilized to generate artificial data, significantly enhancing vessel segmentation performance. The results underscore the efficacy of GAN-generated images in improving segmentation accuracy and the feasibility of training patient-specific models for clinical use.

Moreover, this patient-specific approach empowers clinics to train models with their own data, overcoming data-sharing obstacles prevalent in healthcare machine learning applications.

While our method requires initial on-site labeling of real images by an expert, the process is swift and manageable within standard procedures. Furthermore, while our current work utilizes an ex vivo dataset due to developmental constraints of the fetoscope, in vivo experiments are slated for 2024.

Future work involves conducting in-vivo experiments and exploring applications in other medical procedures where patient-specific training could address data distribution shifts across patients.

## Data Availability

Some of the data used can be found on: https://www.ucl.ac.uk/interventional-surgical-sciences/weiss-open-research/weiss-open-data-server/fetoscopy-placenta-data. Other data from our centers can be made available upon request.

## Footnotes

1 An experiment to determine the effect of various amounts of synthetic data mixed with real data.

2 An experiment to determine whether a network trained on other patients but more data is superior to a patient-specific approach with only minimal data.
